# *Ex vivo* assessment of chemotherapy sensitivity of colorectal cancer peritoneal metastases

**DOI:** 10.1093/bjs/znad066

**Published:** 2023-03-15

**Authors:** Peter H Cashin, Maria Söderström, Kristin Blom, Sara Artursson, Claes Andersson, Rolf Larsson, Peter Nygren

**Affiliations:** Department of Surgical Sciences, Section of Colorectal Surgery, Uppsala University, Uppsala, Sweden; Department of Surgical Sciences, Section of Colorectal Surgery, Uppsala University, Uppsala, Sweden; Department of Medical Sciences, Uppsala University, Uppsala, Sweden; Department of Surgical Sciences, Section of Colorectal Surgery, Uppsala University, Uppsala, Sweden; Department of Medical Sciences, Uppsala University, Uppsala, Sweden; Department of Medical Sciences, Uppsala University, Uppsala, Sweden; Department of Immunology, Genetics and Pathology, Uppsala University, Uppsala, Sweden

## Introduction

Patients with peritoneal metastasis from colorectal cancer (PMCRC) may have a chance of cure when treated with cytoreductive surgery (CRS) combined with heated intraperitoneal chemotherapy (HIPEC)^1–5^.

Choice of chemotherapy for HIPEC has been based on knowledge of its systemic effects, pharmacokinetics, technical feasibility, hyperthermic efficacy enhancement, and tolerance^6–8^. Selection of cancer drugs for treatment based on phenotypical assessment of patient cancer cell drug sensitivity *ex vivo* is one approach to personalized cancer treatment. One technique for this is the fluorometric microculture cytotoxicity assay (FMCA) that has been used in drug development and for the development of personalized cancer medicine^9–16^.

This study investigated whether *ex vivo* assessment of drug sensitivity by the FMCA provides predictive information in terms of peritoneal recurrence-free survival (PRFS) and overall survival (OS) in patients treated with CRS and HIPEC for isolated PMCRC.

## Materials and methods

The patient cohort for this study was from a prospectively maintained institutional database at the Uppsala University Hospital, a tertiary care unit for PMCRC in Uppsala, Sweden. Patients with PMCRC treated with CRS and HIPEC have been registered since 2003. *Ex vivo* drug sensitivity testing using the FMCA started in April 2007. Thus, consecutive patients treated with CRS and HIPEC for PMCRC from April 2007 to October 2018 were considered for providing data for this report. Patients underwent HIPEC with either single-drug oxaliplatin, mitomycin C, or irinotecan, or a combination of oxaliplatin and irinotecan. HIPEC was performed in an open manner according to the coliseum method. Single-drug oxaliplatin was dosed at 350–460 mg/m^2^, and oxaliplatin and irinotecan combined at 360 mg/m^2^ for both drugs. These treatments lasted for 30 min. Mitomycin C was dosed at 35 mg/m^2^ divided into three injections with 50 per cent given at time 0, 25 per cent at 30 min, and 25 per cent at 60 min from the start of HIPEC for a total of 90 min. Follow-up data on PRFS and OS were collected for the final analysis. The Uppsala University ethical committee approved the study (Dnr 2007/237 for tumour sampling and *ex vivo* assessment of drug activity, and Dnr 2013/203 for clinical data collection).

### Fluorometric microculture cytotoxicity assay

The FMCA was performed as described^9,10^. A brief description is included in the supplementary material. For each drug and patient, the *ex vivo* IC_50_ (drug concentration producing a cell survival of 50 per cent of unexposed control) was divided into thirds according to increasing IC_50_ values (1–33 percentile, 34–66 percentile, and 67–100 percentile). Samples in the lowest third of values were denoted sensitive, the middle third as intermediate sensitive, and top third as resistant. In the same way, patients were divided into these groups based on the most active drug used for HIPEC. Since preliminary analyses showed no differences between the sensitive and the intermediate-sensitive groups, they were combined and referred to as sensitive (*Fig. S4*).

### Statistics

Descriptive statistics, univariable/multivariable Cox proportional regression, and Kaplan–Meier curves with the log-rank test were employed. For details, refer to the supplementary material.

## Results

### Demography

In total, 165 patients treated with CRS and HIPEC for PMCRC were identified. A flowchart of the study cohort is provided in *Figure S1*. Seventy-eight patients had evaluable FMCA data, of whom 73 were evaluable for survival analysis (37 per cent men and 63 per cent women; mean age 61 years). Patient characteristics and drug sensitivity data are detailed in *Table S1*.

### Survival analysis

**Fig. 1:**
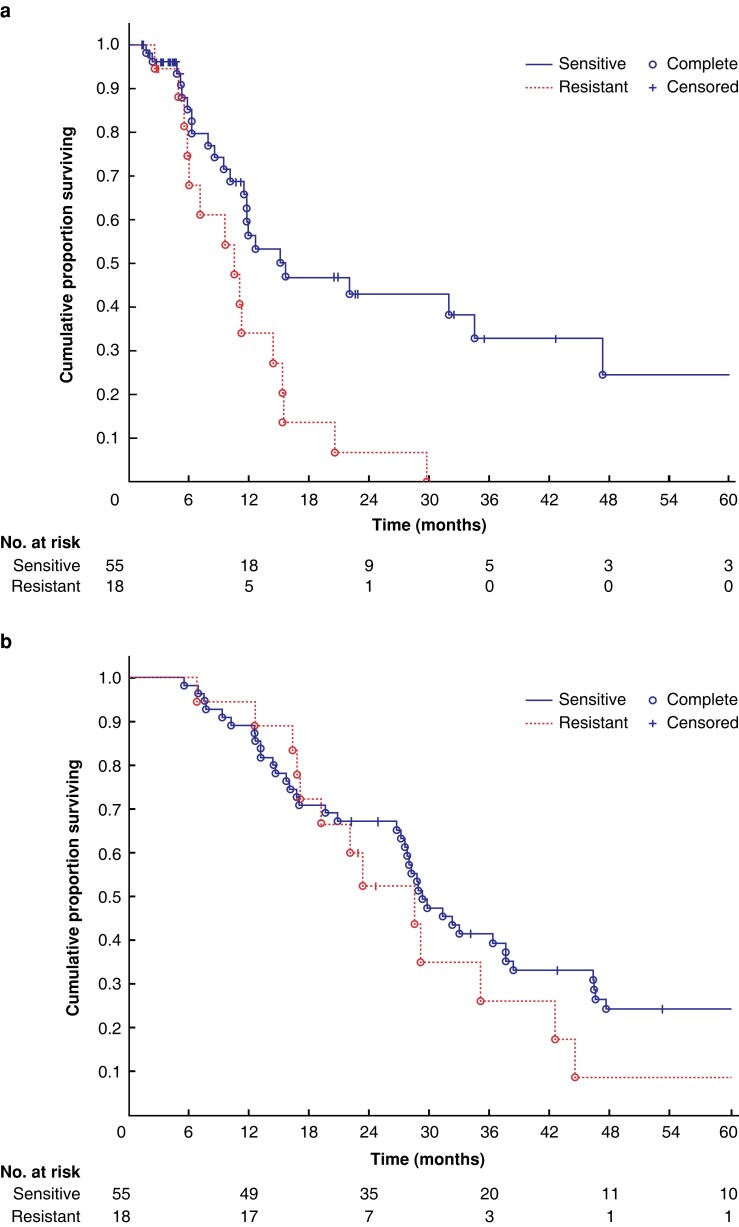
Outcome for all 73 patients with appropriate *ex vivo* data **a** Peritoneal recurrence-free survival for the sensitive and resistant groups (log-rank test *P* = 0.007). **b** Overall survival for the sensitive and resistant groups (log-rank test; *P* = 0.397).

**Fig. 2:**
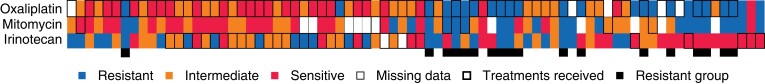
Heat map of individual *ex vivo* chemotherapy testing outcome for 78 patients Each column represents one individual’s *ex vivo* sensitivity to the drugs indicated, with a highlighted frame around the drug(s) used for heated intraperitoneal chemotherapy (HIPEC). Patients having HIPEC with drug(s) scored sensitive or intermediate *ex vivo* were denoted the sensitive group when analysing survival data (*Fig. 1*).

The OS and PRFS of the whole study are detailed in *Fig. S2*. Fifty-five patients undergoing HIPEC with a drug scored as sensitive had a longer PRFS than the 18 undergoing HIPEC with a resistant drug (15.5 (95 per cent c.i. 11.8 to 34.5) *versus* 9.5 months (95 per cent c.i. 6.0 to 11.8); *P* = 0.007, *Fig. 1a*). OS did not differ between groups (*Fig. 1b*). A subgroup analysis that included 36 patients having HIPEC with oxaliplatin only demonstrated a similar pattern for both PRFS (*P* = 0.008) and OS (*P* = 0.232; *Fig. S3*). Univariable/multivariable HRs are reported in *Table S2*. Sensitivity to the drug used for HIPEC remained the only independent prognostic factor (adjusted HR 0.39, 95 per cent c.i. 0.19 to 0.77; *P* = 0.007). Twelve of the 18 patients (67 per cent) having HIPEC with a resistant drug had a drug to which their tumour cells were actually sensitive *ex vivo*, and only six patients had no sensitive option to choose from (*Fig. 2*). Of the 12 with a sensitive alternative, irinotecan was the only option in eight cases, mitomycin only in one case, and both irinotecan and mitomycin in three cases (*Fig. 2*). PRFS according to *ex vivo* drug sensitivity cutoffs is shown in more detail in *Figs S4*, *S5*.

## Discussion

The key finding in this study was that *ex vivo* tumour cell drug activity, as assessed by the FMCA, for the drug(s) used during HIPEC provided significant predictive information for PRFS, the endpoint considered to give the best comparison between the *ex vivo* and *in vivo* situation. However, OS did not statistically significantly differ between patients having HIPEC with sensitive *versus* resistant drugs. This might be due to additional factors impacting on OS, notably systemic disease and its treatment. The sample size may also have been too small to detect differences in OS.

The findings from the current study indicate that tumour cell drug sensitivity is important for the efficacy of HIPEC. This reasonably suggests, indirectly, that HIPEC itself impacts the outcome of CRS + HIPEC for the treatment of PMCRC. This contrasts with findings from the recently published PRODIGE 7 trial, which showed no improvement in outcome with the addition of oxaliplatin-based HIPEC to CRS using single-drug oxaliplatin for 30 min^3^. It was recently demonstrated that neoadjuvant oxaliplatin treatment induces oxaliplatin resistance in cells at the time of CRS^17^. This may be one explanation for the lack of HIPEC benefit in the PRODIGE 7 trial, as all patients were heavily pretreated. In contrast, in Sweden, neoadjuvant therapy is currently only used for downstaging purposes; therefore, approximately half of the patients in our cohort were chemotherapy-naïve, potentially affecting the sensitivity of tumour cells to HIPEC.

*Ex vivo* assessment by the FMCA of tumour sensitivity to chemotherapeutic drugs has previously been reported to provide predictive information on survival in haematological and ovarian malignancies in patients treated systemically^12–14^. The present study expands on this experience and indicates that the FMCA also provides predictive information for the efficacy of HIPEC for PMCRC. Owing to few patients with mitomycin or irinotecan HIPEC, this study mainly provides data for oxaliplatin-based HIPEC. Further studies are needed to evaluate other regimens. One weakness of this study is the PRFS evaluation, which is known to be challenging to identify by radiology if the recurrence is very small. However, this is identical for all patients, and, as such, is not a systematic bias toward any particular treatment group.

Two-thirds of patients who were treated with HIPEC with *ex vivo* resistant drug(s) would have had other drug options to which their tumour cells were sensitive. If patients had received HIPEC with oxaliplatin and irinotecan, the number of patients receiving a sensitive drug would have increased from 66 per cent (48 of 73 patients) to 90 per cent (66 of 73 patients). In Sweden, a randomized clinical trial (EFFIPEC) is planned. HIPEC with single-drug oxaliplatin will be compared to HIPEC with combined oxaliplatin and irinotecan, and with 1 day of 5-fluorouracil early postoperative intraperitoneal chemotherapy.

A future clinical trial on individualized HIPEC could be based on *ex vivo* testing of cancer cells prepared from PMCRC tissue obtained at preoperative laparoscopic staging. One of the strengths of the FMCA test is that it only requires 72 h direct incubation after microdissection and requires about 0.5–1 cm^3^ of tumour tissue to get a successful result. This contrasts with the new organoid technology that requires 7 days or more to get results and goes through a more cumbersome process and is, therefore, more costly^18^^,^^19^. The downside to the FMCA test is that luminal biopsies tend to fail due to bacterial and yeast overgrowth limiting endoscopic tissue sampling. However, organoid technology with long-term culturing allows greater flexibility. Laparoscopic peritoneal sampling during a staging procedure is a perfect fit for the quicker and cheaper FMCA test.

## Supplementary Material

znad066_Supplementary_Data

## Data Availability

Data can be made available upon request to the corresponding author.
