# Supplementary material for: Ex vivo assessment of chemotherapy sensitivity of colorectal cancer peritoneal metastases
Source: Br J Surg. 2023 Mar 15;110(9):1080–3. doi: 10.1093/bjs/znad066 (PMC10416678; doi:10.1093/bjs/znad066)
Supplement: znad066_Supplementary_Data [file znad066_supplementary_data.docx]

**TItle**

**Ex vivo assessment of chemotherapy sensitivity of colorectal cancer peritoneal metastases**

Cashin Peter H^1^, Söderström Maria^1^, Blom Kristin^2^, Artursson Sara^1^, Andersson Claes^2^, Larsson Rolf^2^, Nygren Peter^3^

*1 Dept of Surgical Sciences, Section of Colorectal Surgery, Uppsala University, 751 85 Uppsala, Sweden*

*2 Dept of Medical Sciences, Uppsala University*

*3 Dept of Immunology, Genetics and Pathology, Uppsala University*

**Corresponding author.** Associate Professor Peter Cashin, Akademiska sjukhuset, 75185, Uppsala, Sweden

**ORCID ID;** 0000-0003-3474-9450;

**Twitter** @CashinPtr

**Supplementary Materials - Index**

| **Supplementary Methods** |  |
| --- | --- |
| Surgery and HIPEC regimens | *pag. 2* |
| The FMCA analysis  Patient follow-up and assessment of study clinical endpoints  Statistics | *pag. 2*  *pag. 3*  *pag. 3* |
| **Supplementary Figures and Tables** |  |
| Figure S1 – Flowchart | *pag. 5* |
| Figure S2  Figure S3  Figure S4  Figure S5  Table S1  Table S2 | *pag. 6*  *pag. 7*  *pag. 8*  *pag. 9*  *pag. 10*  *pag. 11* |
|  |  |

**Supplementary Methods**

*Surgery and HIPEC regimens*

The patients underwent surgery according to standard peritonectomy techniques. At the beginning of surgery, the peritoneal cancer index (PCI) score was assessed and formed the basis for proceeding to CRS and HIPEC. Completeness of cytoreduction (CC) was used to evaluate quality of the CRS. CC-0 means macroscopically no tumour is left, CC1 means no tumour larger than 2,5 mm is left.

Following CRS, HIPEC was performed in an open manner according to the coliseum method. Single drug oxaliplatin was dosed at 350-460 mg/m2 and oxaliplatin and irinotecan combined at 360 mg/m2 of both drugs. These treatments lasted 30 min. Mitomycin C was dosed at 35 mg/m2 divided into 3 injections with 50% given at time 0, 25% at 30 min and 25% at 60 min from start of HIPEC for a total of 90 min. After HIPEC, surgery continued with reconstructions, anastomoses and stomas as applicable and the abdomen was closed. During surgery and prior to HIPEC, tumour samples, mostly ranging 1- 5 cm3, from peritoneal metastases were procured and immediately brought to the laboratory for processing and FMCA analysis.

*The FMCA analysis*

Briefly, the tumour samples were finely minced followed by collagenase digestion and gradient centrifugation to extract separated tumor cells. A May-Grünwald Giemsa stained cytospin glass was prepared to assess type and quality of the obtained cells. The cells were then diluted and seeded into the wells of a 384-well microplate at a density of 5 000 cells/well.

Between April 2007 and mid 2013 the cells were seeded onto prefabricated compound plates. After mid 2013 an ECHO 550 drug acoustic dispensing robot (Labcyte Inc., Sunnyvale, CA) transferred cytotoxic drugs from premade drug plates to the 384-well plates to predefined concentrations. One column of wells receives only culture medium (blank wells), and at least 3 wells receive only cells but no drugs (control wells). Drugs were tested in 3 or5 steps of 3, 5- or 10-fold dilutions from the maximum concentrations of of 100 µM for oxaliplatin, 100 µM for mitomycin C and 1000 µM for irinotecan. The drugs were from commercially available clinical preparations.

The culture plates were then incubated at 37°C in humidified atmosphere containing 95% air and 5% CO_2_. After 72 h incubation, the culture medium was washed away and 50 μl/well of a physiological buffer containing 10 μg/ml of the vital dye fluorescein diacetate (FDA) were added to control, experimental and blank wells. After incubation for 30 - 45 min at 37°C, the fluorescence from each well was read in a fluorometer.

Quality criteria for a successful assay were: ≥ 70% tumor cells in the cell preparation prior to incubation and/or on the assay day, a fluorescence signal in control cultures of ≥ five times mean blank values, and a coefficient of variation of cell survival in control cultures of ≤ 30%. The results obtained by the viability indicator FDA are presented as survival index (SI), defined as the fluorescence of the test expressed as a percentage of control cultures, with blank values subtracted.

Concentration-response SI data were used to calculate the 50% inhibitory concentrations, i.e. the drug concentration producing a SI of 50%, (IC_50_). This was done using non-linear regression to a standard sigmoidal dose-response model in GraphPad Prism version 5 for Mac (GraphPad Software, San Diego, CA, USA).

The cut-off limits for sensitive, intermediate and resistant tumour cells is explained in the methods section. Besides this analysis, an alternative analysis using the standard cut-off for the FMCA test results was also performed^10^. In this analysis, ex vivo sensitivity is defined as an IC _50_ value below the median among the patient sample analyzed, intermediate sensitivity from the median up to median plus one standard deviation and resistance as an IC _50_ value above median plus one standard deviation. As only 3 patients ended up in the resistant group in this analysis, the analysis using division into thirds as described in the regular methods section was used to evaluate the study endpoints (Figure S2).

*Patient follow-up and assessment of study clinical endpoints*

All patients were followed-up according to clinical routine which entails computer tomography scans of the thorax and abdomen every 6 months until 2 years after CRS + HIPEC and yearly from 3 to 5 years. Serum tumor markers were taken at the same time intervals as the radiology. The main endpoint of the study was PRFS defined as time from surgery to first radiologically defined recurrence in the peritoneal cavity. As systematic follow-up is only conducted until the first recurrence where new treatment options are implemented, patients with recurrence at other sites than the peritoneum were censored from the time of first non-peritoneal recurrence.

*Statistics*

Descriptive statistics were used with Mann-Whitney U test, Pearson Chi^2^ test, or Fisher’s exact test as applicable for calculation of statistical inference. Univariate Cox proportional regression analysis was used to evaluate potential individual prognostic variables. The multivariable Cox proportional regression analysis included the ex vivo drug sensitivity data as well as four of the most important prognostic indicators from literature: PCI, CC, lymph-node metastases and concomitantly resected liver metastases. Kaplan-Meier curves were used to display PRFS and OS for patients in the sensitive and resistant groups. Kaplan-Meier curves for the alternative IC _50_ cut-off values were added as supplementary results.

**Supplementary Figures and Tables**

Figure S1 – Flowchart

Figure S2 – Overall survival and peritoneal recurrence free survival of the whole cohort (A) and peritoneal recurrence free survival according to each HIPEC regimen (B). Median OS and PRFS for the patients with successful ex vivo data were 29 (95% CI: 27.5-37.5) and 12.6 (95% CI: 11.0-15.5) months, respectively. PRFS seemingly did not differ between the different HIPEC regimens used.


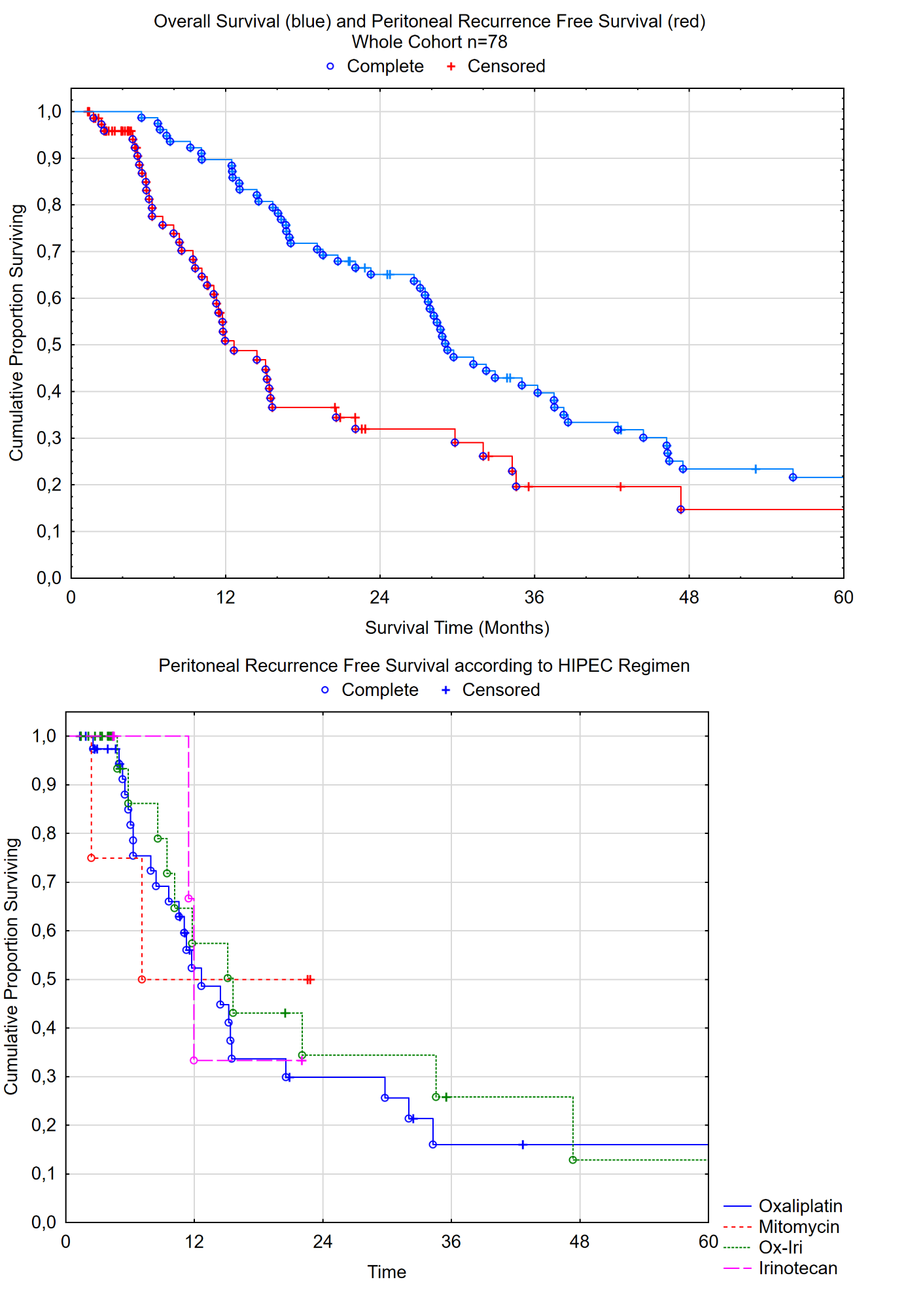


A

B

Figure S3 – Outcome for the subgroup of patients having HIPEC with oxaliplatin only (n=36). A: Peritoneal recurrence-free survival according to sensitive and resistant groups, p=0.0079. B: Overall survival according to sensitive and resistant groups, p=0.23.


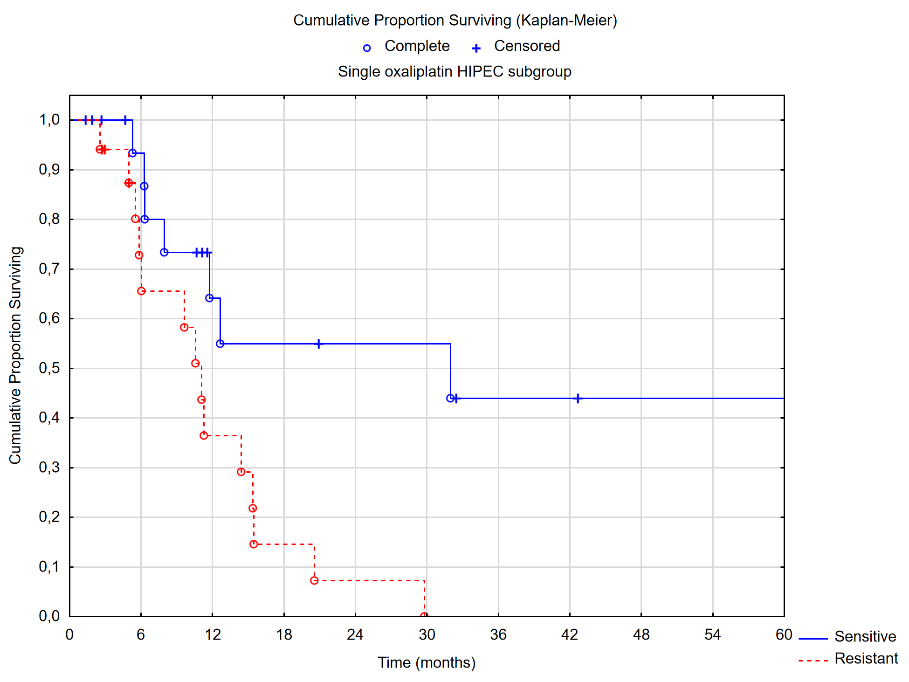


A


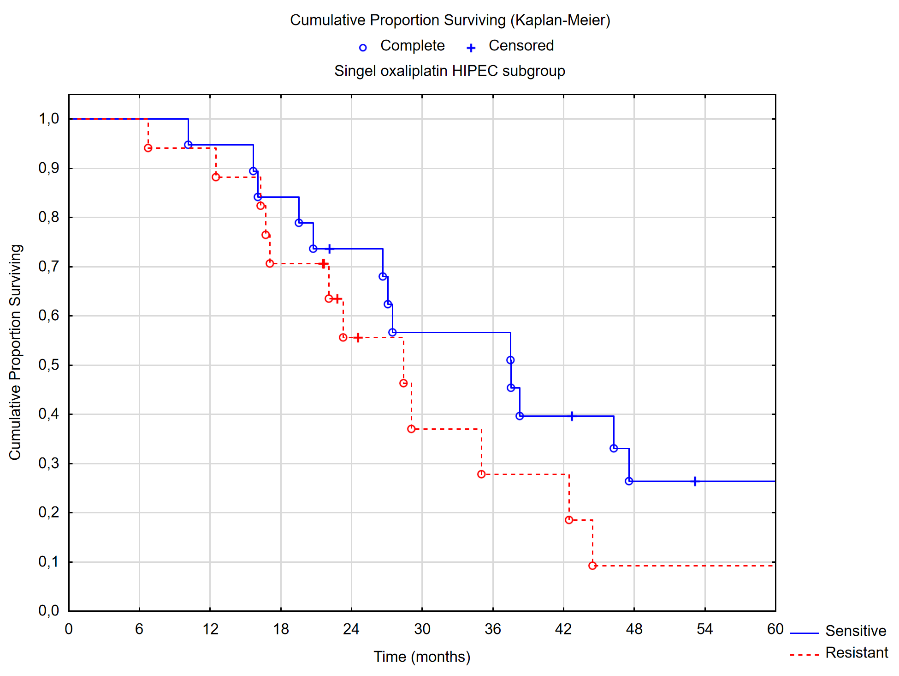


B

Figure S4 – Peritoneal recurrence-free survival according sensitivity cut-offs by three equal percentile divisions – 0-33 percentile, 33-67 percentile and 67-100 percentile. Sensitive group (1) – the patients had HIPEC with at least one drug scoring sensitive ex vivo, n=30. Intermediate group (2) – the patients had HIPEC with the best drug scoring intermediate ex vivo , n=25. Resistant group (3) – the patients had HIPEC with the best drug scoring resistant ex vivo, n=18.


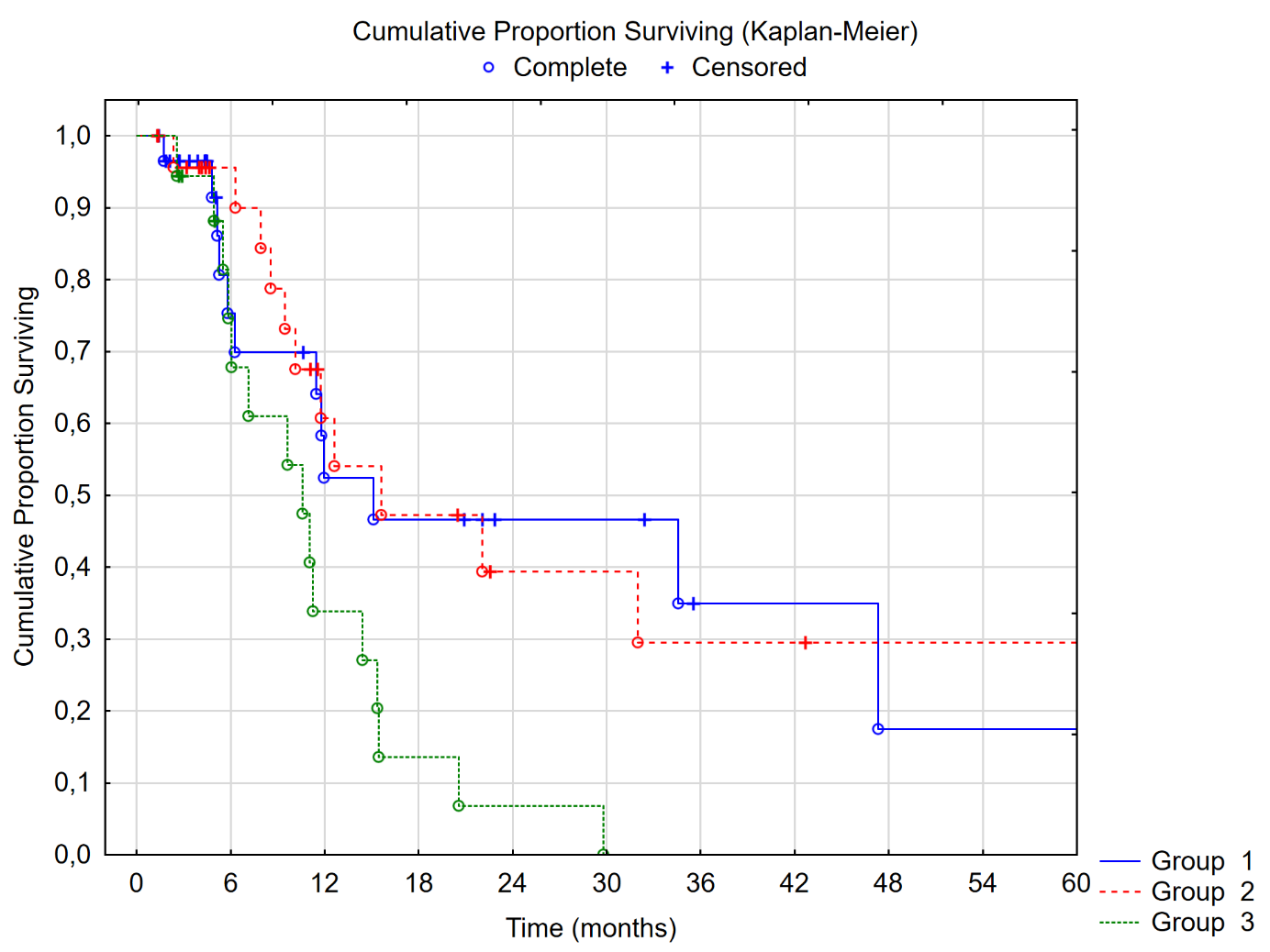


Figure S5 – Peritoneal recurrence-free survival according to sensitivity cut-offs using the median value and the median + 1 standard deviation value. Sensitive group (1) – the patient had HIPEC with at least one drug scoring sensitive ex vivo, n=43. Intermediate group (2) – the patient had HIPEC with the best drug scoring intermediate ex vivo, n=27. Resistant group (3) – the patients had HIPEC with the best drug scoring resistant ex vivo, n=3.


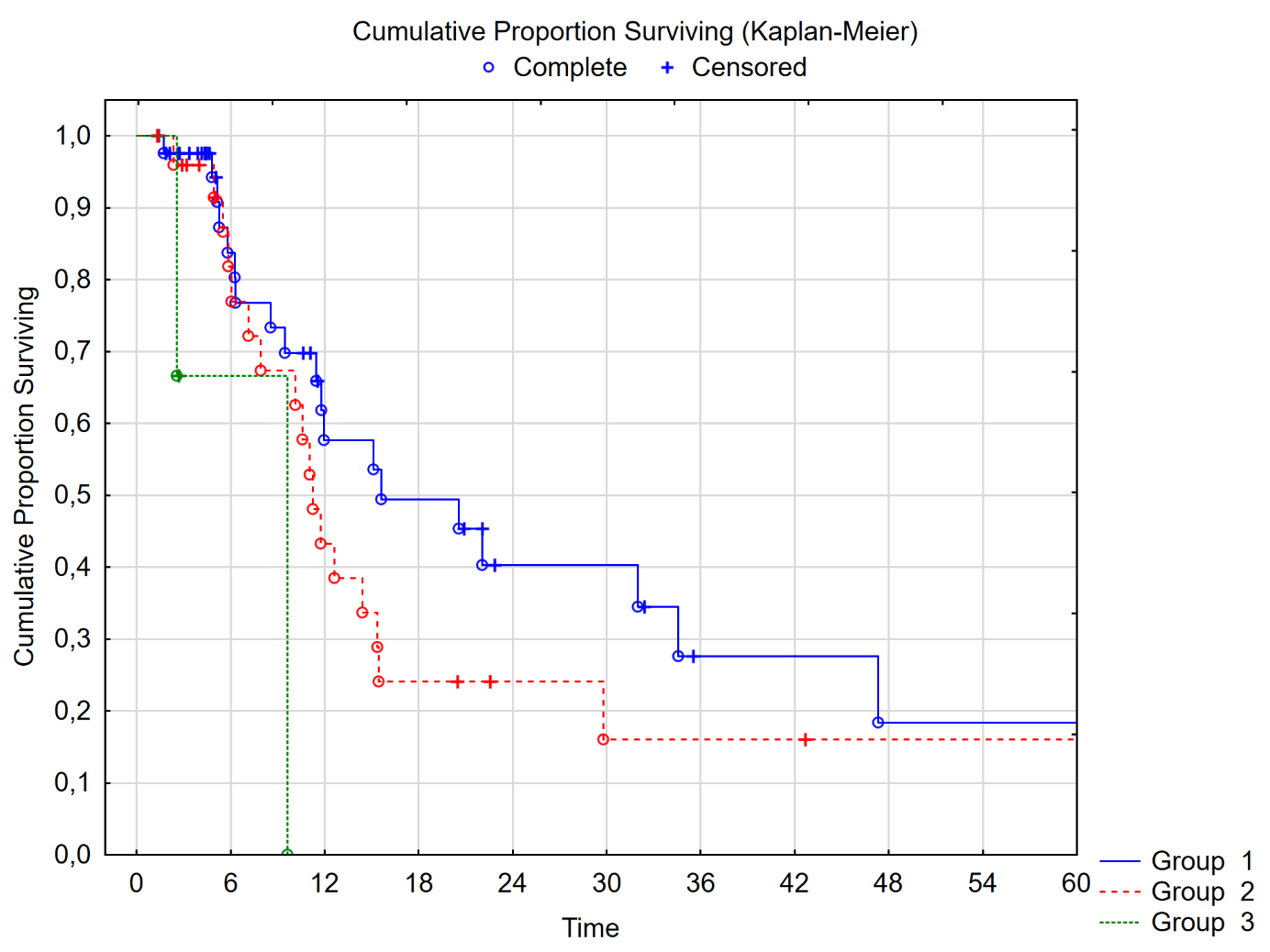


Table S1: Patient characteristics and demography

|  | Whole Cohort  n=78* | Sensitive  Group n=55 | Resistant  Group n=18 | p-value |
| --- | --- | --- | --- | --- |
| Age [median (IQR)] | 61 (49-66) | 59 (47-65) | 63 (56-67) | 0.18 |
| Karnofsky [n (%)]  100  90  80 | 55 (71%)  19 (24%)  4 (5%) | 35 (64%)  16 (29%)  4 (7%) | 16 (89%)  2 (11%)  0 (0%) | 0.57 |
| Gender [n (%)]  Male  Female | 29 (37%)  49 (63%) | 17 (31%)  38 (69%) | 10 (56%)  8 (44%) | 0.47 |
| Primary tumor [n (%)]  Colon  Rectum  Missing | 72 (93%)  5 (6%)  1 (1%) | 52 (95%)  3 (5%)  0 (0%) | 15 (83%)  2 (11%)  1 (6%) | 0.60 |
| Lymph-node positive [n (%)] | 62 (79%) | 42 (76%) | 16 (89%) | 0.83 |
| Poorly differentiated [n (%)] | 27 (35%) | 18 (33%) | 8 (44%) | 0.50 |
| Signet-cell tumor [n (%)] | 14 (18%) | 9 (16%) | 3 (17%) | 0.78 |
| PCI [median (IQR)] | 15 (10-23) | 14 (8-26) | 18 (13-23) | 0.34 |
| CC score [n (%)]  0  1 | 73 (94%)  5 (6%) | 52 (95%)  3 (5%) | 16 (89%)  2 (11%) | 0.90 |
| Liver metastases [n (%)] | 16 (21%) | 11 (20%) | 4 (22%) | 1.00 |
| Preop chemo. for PM disease [n (%)] | 38 (49%) | 31 (56%) | 6 (33%) | 0.33 |
| Postop chemo. [n (%)]  Missing data | 30 (38%)  12 (15%) | 19 (35%)  7 (13%) | 9 (50%)  4 (22%) | 0.10 |
| HIPEC treatment [n (%)]  Oxaliplatin  Oxaliplatin + irinotecan  Mitomycin C  Irinotecan | 40 (51%)  28 (36%)  5 (6%)  5 (6%) | 19 (35%)  28 (51%)  3 (5%)  5 (9%) | 17 (94%)  0 (0%)  1 (6%)  0 (0%) | 0.0012 |
| IC_50_ oxaliplatin µM [median (IQR)] n=74 | 35.9 (13.0-90.0) | 23.2 (9.8-44.6) | 147 (90-356) | <0.0001 |
| IC_50_ mitomycin µM [median (IQR)] n=68 | 18.0 (6.0-67.3) | 9.2 (4.5-29.8) | 156 (32-321) | <0.0001 |
| IC_50_ irinotecan µM [median (IQR)] n=78 | 47.4 (14.0-98.1) | 50.0 (17.9-86.6) | 42 (8-103) | 0.51 |

*Since 5 patients had no IC_50_ value for the HIPEC drug administered, they could not be evaluated as sensitive or resistant.

Table S2 - Univariate and multivariable Cox regression analysis for peritoneal recurrence-free survival.

|  | Univariate analysis | p-value | Multivariable analysis | p-value |
| --- | --- | --- | --- | --- |
| Age | 1.01 (0.99-1.04) | 0.33 |  |  |
| Karnofsky  100  90  80 | Reference  1.49 (0.70-3.18)  1.14 (0.34-3.77) | 0.46  0.90 |  |  |
| Gender  Male  Female | Reference  0.69 (0.37-1.29) | 0.25 |  |  |
| Primary tumor  Colon  Rectum | 2.30 (0.31-17.0)  Reference | 0.70 |  |  |
| Lymph-node positive | 1.40 (0.61-3.18) | 0.43 | 1.27 (0.48-3.35) | 0.63 |
| Poorly differentiated | 1.66 (0.85-3.25) | 0.99 |  |  |
| Signet-cell tumor | 1.03 (0.49-2.17) | 0.94 |  |  |
| PCI | 1.03 (0.99-1.07) | 0.057 | 1.03 (0.99-1.07) | 0.098 |
| CC score  0  1 | 0.57 (0.20-1.60)  Reference | 0.28 | 0.90 (0.30-2.76)  Reference | 0.86 |
| Liver metastases | 0.90 (0.40-2.03) | 0.79 | 1.06 (0.43-2.62) | 0.90 |
| Preop chemotherapy for PM disease | 0.82 (0.44-1.54) | 0.54 |  |  |
| Postop chemotherapy | 0.79 (0.40-1.55) | 0.49 |  |  |
| HIPEC treatment  Oxaliplatin  Oxaliplatin + irinotecan  Mitomycin C  Irinotecan | Reference  0.79 (0.39-1.63)  0.76 (0.10-5.97)  0.81 (0.19-3.47) | 0.85  0.89  0.97 |  |  |
| IC_50_ oxaliplatin | 1.00 (1.00-1.00) | 0.39 |  |  |
| 0.9-58.8 µM IC_50_*  58.8-1826 µM IC_50_* | 0.69 (0.39-1.24)  Reference | 0.21 |  |  |
| IC_50_ mitomycin | 1.00 (1.00-1.00) | 0.07 |  |  |
| 0.4-37.6 µM IC_50_*  37.6-2834 µM IC_50_* | 0.58 (0.31-1.10)  Reference | 0.096 |  |  |
| IC_50_ irinotecan | 1.00 (1.00-1.00) | 0.39 |  |  |
| 1.7-76.3 µM IC_50_*  76.3-2697 µM IC_50_* | 0.87 (0.50-1.53)  Reference | 0.64 |  |  |
| Sensitivity according to HIPEC drug given^#^  Sensitive group  Resistant group | 0.38 (0.19-0.75)  Reference | 0.0051 | 0.39 (0.19-0.77)  Reference | 0.0067 |

*0-67^th^ percentile vs 67-100^th^ percentile, ^#^Sensitive group included patients that were treated with at least one HIPEC drug that fell into the sensitive two-thirds category, while the resistant group only received HIPEC drugs that fell into the top one-third resistant category
